# Combination treatment of radiofrequency ablation and peptide neoantigen vaccination: Promising modality for future cancer immunotherapy

**DOI:** 10.3389/fimmu.2022.1000681

**Published:** 2022-09-29

**Authors:** Jiawei Shou, Fan Mo, Shanshan Zhang, Lantian Lu, Ning Han, Liang Liu, Min Qiu, Hongseng Li, Weidong Han, Dongying Ma, Xiaojie Guo, Qianpeng Guo, Qinxue Huang, Xiaomeng Zhang, Shengli Ye, Hongming Pan, Shuqing Chen, Yong Fang

**Affiliations:** ^1^ Sir Run Run Shaw Hospital, Zhejiang University School of Medicine, Hangzhou, China; ^2^ Hangzhou Neoantigen Therapeutics Co., Ltd., Hangzhou, China; ^3^ College of Pharmaceutical Sciences, Zhejiang University, Hangzhou, China; ^4^ Vancouver Prostate Centre, University of British Columbia, Vancouver, BC, Canada; ^5^ Hangzhou AI-Force Therapeutics Co., Ltd., Hangzhou, China; ^6^ Zhejiang California International Nanosystems Institute, Zhejiang University, Hangzhou, China; ^7^ School of Chemistry and Molecular Biosciences, The University of Queensland, Brisbane, QLD, Australia; ^8^ Hangzhou AI-Nano Therapeutics Co., Ltd., Hangzhou, China; ^9^ Shulan (Hangzhou) Hospital, Hangzhou, China; ^10^ ZJU-Hangzhou Global Scientific and Technological Innovation Center, Hangzhou, China

**Keywords:** neoantigen vaccine, radiofrequency ablation, immune checkpoint inhibition, cancer, immunotherapy

## Abstract

**Background:**

The safety and immunogenicity of a personalized neoantigen-based peptide vaccine, iNeo-Vac-P01, was reported previously in patients with a variety of cancer types. The current study investigated the synergistic effects of radiofrequency ablation (RFA) and neoantigen vaccination in cancer patients and tumor-bearing mice.

**Methods:**

Twenty-eight cancer patients were enrolled in this study, including 10 patients who had received RFA treatment within 6 months before vaccination (Cohort 1), and 18 patients who had not (Cohort 2). Individualized neoantigen peptide vaccines were designed, manufactured, and subcutaneously administrated with GM-CSF as an adjuvant for all patients. Mouse models were employed to validate the synergistic efficacy of combination treatment of RFA and neoantigen vaccination.

**Results:**

Longer median progression free survival (mPFS) and median overall survival (mOS) were observed in patients in Cohort 1 compared to patients in Cohort 2 (4.42 and 20.18 months vs. 2.82 and 10.94 months). The results of *ex vivo* IFN-γ ELISpot assay showed that patients in Cohort 1 had stronger neoantigen-specific immune responses at baseline and post vaccination. Mice receiving combination treatment of RFA and neoantigen vaccines displayed higher antitumor immune responses than mice receiving single modality. The combination of PD-1 blockage with RFA and neoantigen vaccines further enhanced the antitumor response in mice.

**Conclusion:**

Neoantigen vaccination after local RFA treatment could improve the clinical and immune response among patients of different cancer types. The synergistic antitumor potentials of these two modalities were also validated in mice, and might be further enhanced by immune checkpoint inhibition. The mechanisms of their synergies require further investigation.

**Clinical trial registration:**

https://clinicaltrials.gov/, identifier NCT03662815.

## Introduction

Neoantigens are a type of tumor-specific antigens that are generated by non-synonymous mutations (1). During the past five years, neoantigen-based personalized cancer vaccines have thrived as a result of recent advancement in sequencing technologies and immunoinformatic algorithms. However, neoantigen vaccination alone might be inefficient to elicit robust responses to eliminate tumors, due to the complicated tumor immunosuppressive microenvironment (2). Thus, a variety of different strategies have been explored in both preclinical and clinical settings to improve the efficacy of neoantigen vaccines (3). For example, vaccine adjuvants such as toll-like receptors (TLRs) have been administered with neoantigen vaccines to enhance their anticancer efficacy. Besides, various delivery approaches are being investigated to improve the unfavorable *in vivo* pharmacokinetics of neoantigens, and boost the insufficient immune responses resulting from the discrepant physicochemical properties of neoantigens (2, 4–8). Currently, it seems promising to couple neoantigen vaccines with other modalities that can modify or revert the immunosuppressive TME, such as immune checkpoint inhibition (ICI) (3–7, 9, 10). Up to date, a variety of trials have been launched to investigate the efficacy and underlying mechanisms of the combination of ICIs with neoantigen vaccines (NCT03715985, NCT03422094, NCT02287428, NCT02950766, NCT03380871 and NCT02897765).

Radiofrequency ablation (RFA) has been commonly utilized in clinics as a minimally invasive treatment, increasing local temperature to induce coagulative necrosis of tumor. Besides, it has been demonstrated that RFA can activate the host’s immune system through different mechanisms (11). Firstly, cell death resulted from RFA-induced hyperthermic injury and coagulative necrosis usually leads to the release of both intracellular antigens and damage-associated molecular patterns (DAMPs) such as heat shock proteins, mobility group protein B1, as well as nucleic acids (DNA and RNA), which can be endocytosed and processed by dendritic cells (DCs) to elicit a systemic immune response (12–14). Secondly, RFA can modulate tumor microenvironment (TMB) by upregulating the expression of immunostimulating factors including interferon-γ (IFN-γ), tumor necrosis factor alpha (TNF-α), interleukin (IL)-1β, IL-8 and IL-2, and inhibiting the expression of immunosuppressive soluble IL-2 receptor and hepatocyte growth factor (12). Moreover, after RFA treatment, it is common to observe the accumulation of local tumor-infiltrating lymphocytes and peripheral circulation lymphocytes (12), together with the decease of the proportions of regulatory T cells, tumor-associated macrophages and neutrophiles (14), mounting systemic antitumor responses. However, despite the above anticancer advantages, RFA alone may still be insufficient to prevent cancer progression or inhibit cancer recurrence due to many factors such as the inefficient antigen presentation by DCs (13, 15). Recently, the synergistic antitumor activity of RFA and ICI has been reported in both preclinical and clinical settings (11, 15, 16). Also, PD-L1, PD-1 and LAG3 were found to be upregulated in distant non-RFA tumors after RFA treatment, indicating the synergy between RFA and ICI (14, 15). Several clinical trials have been launched to explore the efficacy and mechanisms of the combination of RFA with ICI (NCT01853618; NCT02821754; NCT03939975) (12).

As described above, both neoantigen vaccines and RFA require synergetic therapeutic modality to further enhance their efficacy to prevent tumor progression or inhibit tumor recurrence. The fact that RFA could elicit pre-vaccination response (10, 17), encourages the combination of RFA with neoantigen vaccines to translate into clinical benefits. Since both RFA and neoantigen vaccination could synergize with ICIs; it would be worthwhile to investigate the synergies between RFA, neoantigen vaccines and ICIs in combating tumors. We report here the promising results of RFA treatment prior to neoantigen vaccination in patients of different cancer types, as well as the synergistic antitumor effects of combinational RFA, neoantigen vaccination and ICIs in a mouse colorectal tumor model.

## Methods

### Clinical trial design and patient characteristics

Patients aged ≥ 18 with different cancer types including colon cancer, melanoma, lung cancer, pancreatic cancer, biliary tract cancer, ovarian cancer, breast cancer, gastric cancer, parotid cancer, and adrenal sebaceous adenocarcinoma were enrolled in a single-arm, open-label and investigator-initiated phase I study at Sir Run Run Shaw Hospital, School of Medicine, Zhejiang University in China (NCT03662815). Eligible patients had a baseline Eastern Cooperative Oncology Group (ECOG) performance status of 0-1. The primary endpoints of this study were safety and feasibility, and the secondary endpoint was efficacy based on progression-free survival (PFS), overall survival (OS), and neoantigen-specific immune response. For all enrolled patients, the treatment scheme and clinical response of personalized vaccines were summarized in **Supplementary Table S1**. Colorectal patients receiving RFA and neoantigen vaccines in our study were retrospectively reviewed and compared to colorectal patients (RFA colorectal group) who received only RFA at Sir Run Run Shaw Hospital affiliated with Zhejiang University School of Medicine between 2017 and 2020, for their OS. Patients with similar characteristics to our patients including age, sex distribution, tumor metastases, prior systemic therapies and RFA treatments were selected for comparison.

Clinical assessment, monitoring, and follow-up including physical examination, ECOG performance, vital sign, blood test as well as urinalysis were conducted on a regular basis according to the follow-up plan. Imaging tests at baseline and approximately every 8 weeks post-vaccination were arranged for each patient to assess the clinical efficacy. Enzyme-linked immunospots (ELISpot) assay, T-cell receptor (TCR) sequencing, and flow cytometry analyses on cytokines and T-cell subsets were performed pre- and post-vaccination to evaluate the specific immune response (**Supplementary Methods**). All tumors were assessed by investigators according to RECIST v1.1 criterion at baseline and approximately every 8 weeks thereafter. Each patient was monitored during vaccine treatment and followed up every 3 months after the discontinuation of treatment. Treatment-related adverse events (AEs) were recorded and graded for safety evaluation according to the National Cancer Institute Common Terminology Criteria for Adverse Events (version 4.03) throughout the whole treatment. This study was approved by Institutional Review Board and Independent Ethics Committee, and conducted in accordance with the Declaration of Helsinki and the International Conference on Harmonization Guidelines for Good Clinical Practice. All patients had given written informed consent before the initiation of any study procedures. Signed permission to disclose their personal health information (PHI) was collected.

### Vaccines and immunization schedule

Whole-exome sequencing was performed for each patient (**Supplementary Methods**; **Supplementary Table S2**). Personalized peptide vaccines based on identified neoepitopes as well as HLA typing of each patient, were designed using our in-house pipeline iNeo-Suite (**Supplementary Methods**; **Supplementary Table S3** and **Supplementary Table S4**). All peptides were manufactured through solid-phase peptide synthesis at GMP-like standard (bacteria-free, less than 10 EU/mg endotoxin, > 95.0% purity) (**Supplementary Table S5**). Water insoluble peptides were removed from the formulation of iNeo-Vac-P01, as auxiliary solvents such as dimethyl sulfoxide (DMSO) were not applicable due to ethical concerns.

iNeo-Vac-P01 comprises of 5~20 peptides with the length ranging from 15 to 35 amino acids. The customized peptides were pooled into 3~4 groups according to their HLA typing, affinity, and allele frequency, and delivered subcutaneously (s.c.) to both sides of upper arms and para-umbilical area at a dose of 100 or 300 μg/peptide per 1mL injection (**Supplementary Table S1**). For vaccination, iNeo-Vac-P01 was administered during the first month on day 1, 4, 8, 15 and 22 as prime. Boosts were given to each patient on day 78 and 162, and every 2 to 3 months afterwards until disease progression was found. Another batch of vaccines was designed and administered for several patients, as they had an extra puncture biopsy on their new lesions during the treatment and had good treatment compliance. Prior to each vaccination, 800 μL granulocyte-macrophage colony-stimulating factor (GM-CSF) was injected (s.c.) as adjuvant at a concentration of 50 μg/mL nearby each injection site of iNeo-Vac-P01.

### Animal study

Seventy BALB/c female mice aged 6-8 weeks were purchased from Shanghai Slac Laboratory Animal Co. Ltd., and implanted with either bilateral or unilateral tumors. The mouse colon cancer cell line CT26 and breast cancer cell line 4T1 were obtained from Center for New Drug Safety Evaluation and Research, China Pharmaceutical University. Adherent tumor cells were cultured as described in **Supplementary Methods**. To obtain bilateral tumors, CT26 or 4T1 tumors were subcutaneously injected at the density of 1×10^6^/100 μL on the left flank, while CT26 tumor was injected at the density of 7.5×10^4^/100 μL on the right flank. Unilateral tumor model was established by subcutaneous injection of CT26 cells at a density of 7.5×10^4^/100 μL on the right flank.

Twenty mice bearing CT26 unilateral tumor were randomly and evenly divided into 2 groups, and then treated with PBS (PBS group), and iNeo-Vac-P01 (Vac group), respectively. Similarly, another 4 groups of mice bearing CT26 bilateral tumors were treated with RFA, RFA in combination with anti-PD-1 (*InVivo*MAb anti-mouse PD-1, Bio X Cell, Lebanon, USA) (RFA+anti-PD-1), RFA in combination with Vac (RFA+Vac), and RFA in combination with both Vac and anti-PD-1 (RFA+Vac+anti-PD-1), respectively. Only tumors on the left flank were ablated. In addition, another group of mice (n=10) were implanted with bilateral heterogenous tumors (CT26 tumor on the right flank, and 4T1 tumor on the left flank), and received RFA on the left flank to study the abscopal effect as a control group. Mice bearing bilateral tumors were scheduled to receive RFA treatment 6 days post tumor inoculation, where the size of the left flank tumors reached around 6 mm in diameter. RFA was performed using a clinically available ablation device consisted of a radiofrequency energy generator (STARmed, RF generator VRS01) and a 17-gauge single ablation electrode with an active tip of 1 cm (STARmed, 18-07s07f). The tip was inserted orthogonally into the center of the tumor, with the energy titrated to ensure a tip temperature around 70°C for 3.5 to 4.5 minutes depending on the actual tumor size.

CT26-specific neoantigens were identified, prioritized, and then designed into peptide vaccines (**Supplementary Methods**, **Supplementary Table S12**). iNeo-Vac-P01 (100 μL, 0.1 mg/peptide) was administered s.c. to mice on day 8, 9, 12, 16, 21 and 28 after tumor inoculation. Anti-PD-1 antibody (100 μL, 200 μg/mL) was administered to mice intraperitoneally on day 14, 17, 20 and 23. Potential biomarkers were investigated using peripheral blood samples for flow cytometry. Both spleen and peripheral blood samples were tested using ELISpot assay to evaluate the neoantigen-specific immune response (**Supplementary Methods**).

All experiments were approved by Laboratory Animal Management and Ethics Committee of ZCMU (approval number: IACUC-20210301-02) and performed in accordance with the guidelines of Animal Ethical and Welfare Committee of ZCMU. Anesthesia was performed by isoflurane inhalation. All tumors were implanted into the mice’s flanks to minimize distress and negative effects on normal body functions. When the tumor size reached a maximum diameter of 20 mm or mice started to show signs of suffering such as reluctance to move, hunched posture, ≥ 20% weight loss, or body condition scored 2/5 or less, euthanasia was performed. At the end of this study, all mice were euthanized by CO_2_ inhalation.

### Statistical analysis

The data used in the analyses of safety and clinical effects were collected from patients who received at least one dose of iNeo-Vac-P01. Descriptive statistics were applied to determine the characteristics of baseline and assess the safety profile of vaccine. Disease control rate (DCR) was defined as the proportion of complete response (CR), partial response (PR) and stable disease (SD) for best clinical response. Standard RECISTv1.1 guideline was applied for the analysis of clinical response. The Kaplan-Meier curve of patients was plotted with GraphPad Prism 5 (v5.01) using logrank test.

## Results

### Patient demographics

A total of 28 patients with different tumor types, including colon cancer, melanoma, lung cancer, pancreatic cancer, biliary tract cancer, ovarian cancer, breast cancer and others were enrolled in this study from February 7^th^, 2018. All data in this study were collected before May 1^st^, 2021. Although patients were not initially randomized into separate cohorts, distinct differences in clinical response and immune response were found between Cohort 1 (RFA+Vac group, n=10) and Cohort 2 (Vac group, n=18) after retrospective analysis. Patients in Cohort 1, referred as RFA+Vac patients, had received single or multiple RFA treatments within 6 months before they received their first dose of personalized vaccine; while patients in Cohort 2, referred as Vac patients, had not. The patient demographics, baseline disease characteristics, and previous treatments were summarized in **Table 1**, indicating that amongst RFA+Vac patients, 4 (40%), 5 (50%) and 1 (10%) patients had liver metastases, both liver and lung metastases, and bone metastases, respectively. Personalized peptide vaccine, iNeo-Vac-P01, was successfully manufactured and scheduled for all patients. The treatment scheme and clinical response of each patient were presented in **Supplementary Table S1**. No significant difference was found for the lines of prior systematic therapy between RFA+Vac and Vac groups.

**Table 1:** Demographics and characteristics at baseline.

Characteristics		RFA+Vac Patients (N = 10)		Vac Patients (N = 18)
**Age category-no. (%)**					
<65 yrs.		7 (70.00)		14 (77.78)
≥ 65 yrs.		3 (30.00)		4 (22.22)
**Sex-no. (%)**					
Male		5 (50.00)		9 (50.00)
Female		5 (50.00)		9 (50.00)
**Metastatic sites-no. (%)**					
Liver		4 (40.00)		1 (5.56)
Lung		0		4 (22.22)
Liver & lung		5 (50.00)		5 (27.78)
Bone		1 (10.00)		2 (11.11)
**ECOG performance-status score-no. (%)**				
0		5 (50.00)		2 (12.50)
1		5 (50.00)		14 (87.50)
**Radiofrequency ablation-no. (%)**					
Lung		1 (10.00)		0
Lung & liver		4 (40.00)		0
Liver		5 (50.00)		0
**Lines of prior systematic therapy-no. (%)**					
2		3 (30.00)		6 (33.33)
≥3		7 (70.00)		12 (66.67)
**Tumor type-no. (%)**					
Colon Cancer		5 (50.00)		3 (16.67)
Melanoma		1 (10.00)		3 (16.67)
Lung Cancer		1 (10.00)		3 (16.67)
Pancreatic Cancer		1 (10.00)		2 (11.11)
Biliary Tract Cancer		1 (10.00)		1 (5.56)
Ovarian Cancer		0		2 (11.11)
Breast Cancer		0		2 (11.11)
Others		1 (10.00)		2 (11.11)

### Safety and tolerability of iNeo-Vac-P01 vaccination

**Figure 1:**
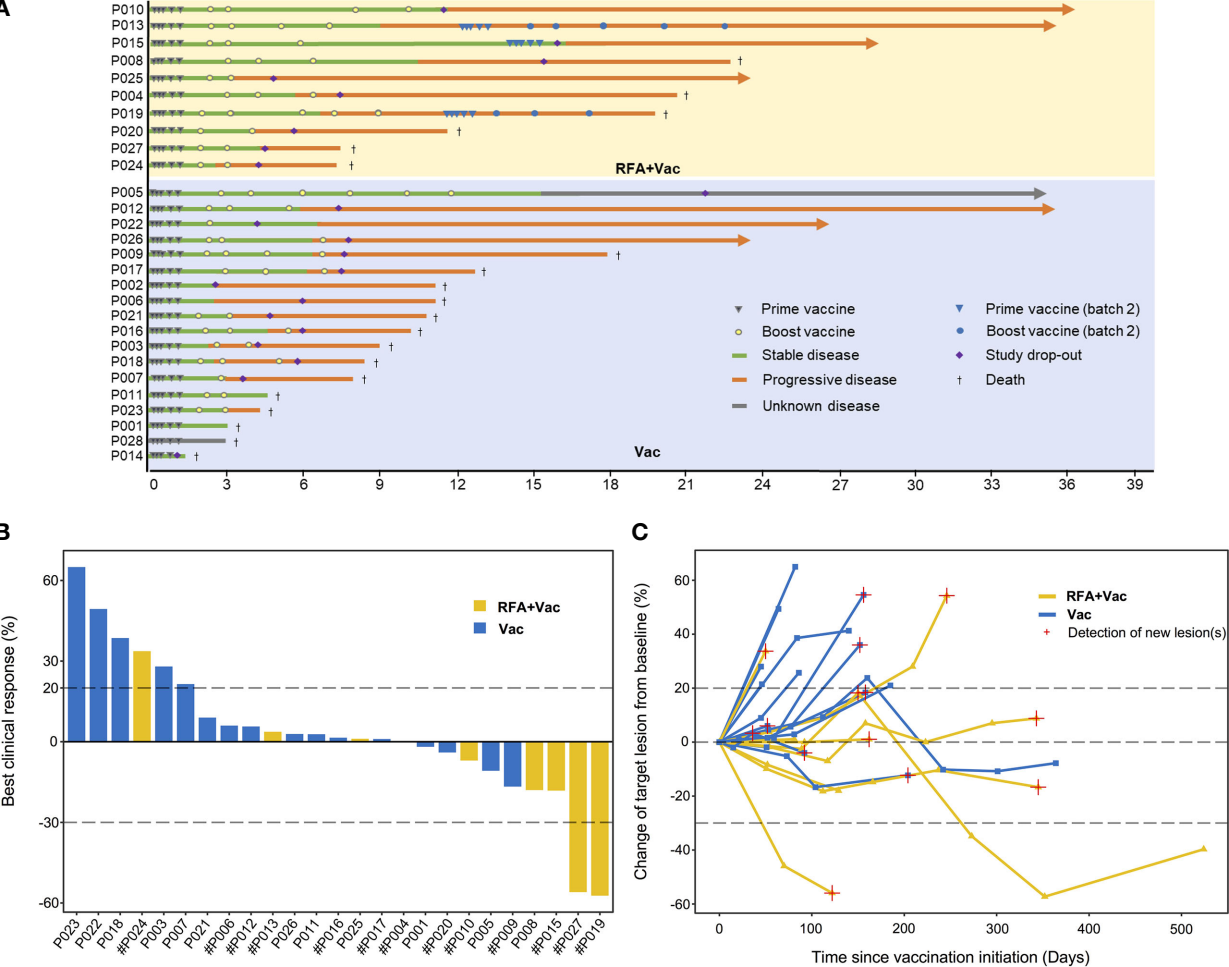
Better clinical response in RFA+Vac patients upon receiving iNeo-Vac. **(A)** Swimmer plots of RFA+Vac patients (with yellow background) and Vac patients (with blue background); **(B)** Waterfall plots of RFA+Vac patients (shown in yellow) and Vac patients (shown in blue). Dashed line above 20% or below -30% indicate 20% increase (≥ 20% is considered as progressive disease) or 30% reduction (≤ -30% is considered as partial response) of the sum of the longest diameters of the tumors, respectively. # suggested that new lesion(s) was found; **(C)** Spider plots of RFA+Vac patients and Vac patients. The “best clinical response” in panel b was defined as the largest reduction of target lesion in size during treatment.

All patients received at least 5 doses of vaccines as prime doses, and 1 - 6 boost doses, except for Patient P014 who dropped out of study before the 5^th^ prime vaccination. A second batch of iNeo-Vac-P01 was scheduled for patients P013, P015 and P019 **(** **Figure 1A**), and clinical evaluation on PFS of these patients was based on the efficacy of vaccination during the first batch only. The treatment was generally well tolerated, with most AEs being graded grade 1-2 (**Table 2**). The most commonly reported treatment-related toxicities were fatigue, fever and muscle soreness. No treatment-related serious adverse events (SAEs) or death was reported. All AEs were reversible without special nursing. The types of treatment-related AEs were considered unrelated to the types of cancer.

**Table 2:** Treatment related adverse events in all treated patients.

	RFA+Vac Patients (n = 10)				Vac Patients (n = 18)			
	Grade 1-2		Grade 3-4		Grade 1-2		Grade 3-4	
	No.	%	No.	%	No.	%	No.	%
Any AE	8	80.00	1	10.00	9	50.00	1	5.55
Fever	4	40.00	0	0	2	11.11	0	0
Fatigue	7	70.00	0	0	6	33.33	0	0
Chill	2	20.00	0	0	0	0	0	0
Emesis	1	10.00	0	0	1	5.55	0	0
Muscle soreness	4	40.00	0	0	1	5.55	0	0
Injection site reaction	0	0	0	0	1	5.55	0	0
Dizziness	0	0	0	0	1	5.55	0	0
Nausea	1	10.00	0	0	0	0	0	0
Upper gastrointestinal haemorrhage	1	10.00	0	0	0	0	0	0
Weight loss	0	0	0	0	1	5.55	0	0
Acute allergy	0	0	1	10.00	0	0	1	5.55

### Neoantigen vaccination showed better clinical response in RFA+Vac patients

In this study, at least one target lesion was evaluated for each patient. For the 9 evaluable RFA+Vac patients, 11.11% (1/9) displayed PR, 77.78% (7/9) displayed SD and 11.11% (1/9) displayed progression disease (PD); while for the 16 evaluable Vac patients, 68.75% (11/16) displayed SD and 31.25% (5/16) displayed PD (**Figure 1** and **Supplementary Table S11**). Three patients (P002, P003 and P014) were excluded from due to the development of new lesions before their first post-treatment follow-up assessment. Overall, tumor reduction was observed in 55.56% (5/9) RFA+Vac patients and only 25.00% (4/16) Vac patients (**Figures 1B, C**).

**Figure 2:**
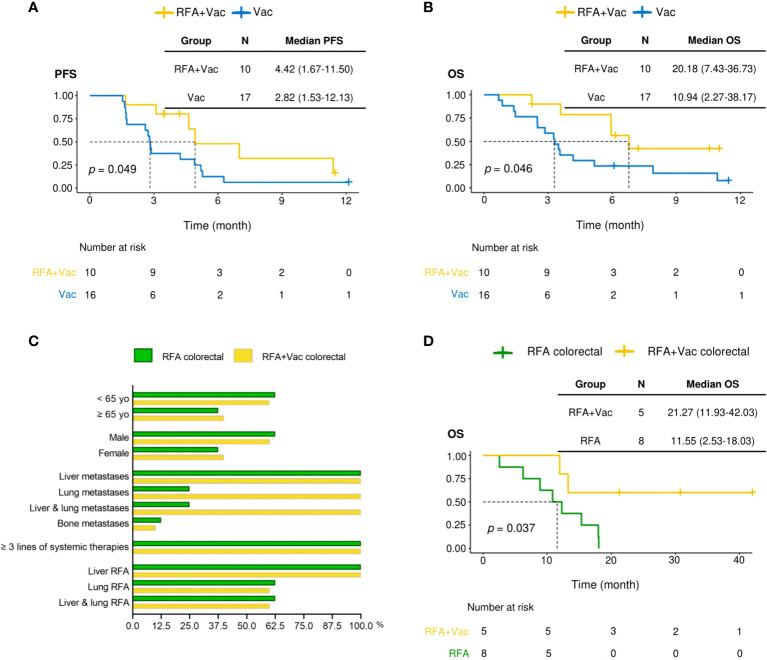
RFA+Vac patients showed better progression-free survival (PFS) and overall survival (OS). **(A)** Progression-free survival of Vac patients (shown in blue) and RFA+Vac patients (shown in yellow). **(B)** Overall survival of Vac patients (blue) and RFA+Vac patients (yellow). **(C)** Characteristics of patients in RFA+Vac colorectal group, in comparison to that of patients in RFA colorectal group. **(D)** Overall survival of RFA-pretreated colorectal cancer patients (RFA+Vac colorectal) (yellow) in this study, in comparison to RFA-treated colorectal cancer patients (RFA colorectal) (green) during the same period at Sir Run Run Shaw Hospital. Longer OS was observed for patients receiving additional neoantigen vaccination (*p* = 0.037). Survival data were compared by logrank test.

Nine RFA+Vac patients and eighteen Vac patients were followed up for progression free survival (PFS) and overall survival (OS) **(** **Figure 2**). One patient (P014) in RFA+Vac group was excluded from this assessment due to study drop-out before the completion of prime vaccination. The rest patients all received ≥ 5 doses of vaccines. The median PFS (mPFS) of RFA+Vac patients was 4.42 months (1.67 – 11.50 months); while Vac patients 2.82 months (1.53 – 12.13 months) (**Figure 2A**). The median OS (mOS) of RFA+Vac patients was 20.18 months (7.43 – 36.73 months), longer than 10.94 months (2.27 – 38.17 months) for Vac patients (**Figure 2B**). Differences in mPFS and mOS between RFA+Vac and Vac groups were both statistically significant (*p* < 0.05).

### Combination treatment benefits colorectal patients in survival

To further evaluate whether the application of personalized vaccine iNeo-Vac-P01 could improve the clinical response of patients after RFA treatment, the OS of colorectal cancer patients receiving combination treatment of RFA and iNeo-Vac-P01 in this trial (RFA+Vac colorectal group) (n=5) were compared to that of colorectal cancer patients receiving RFA treatment alone (RFA colorectal group) (n=8) during the same period. By comparing the patients’ baseline characteristics, including age, sex distribution, tumor metastases (excluding lung metastases), prior systemic treatment as well as RFA treatment, no significant differences were found between RFA+Vac colorectal and RFA colorectal groups (**Figure 2C**). The mOS of RFA+Vac colorectal patients was 21.27 months (11.93-42.03 months), while RFA colorectal patients 11.55 months (2.53-18.03 months) (**Figure 2D**), indicating the efficacy of iNeo-Vac-P01 vaccination after RFA in improving colorectal patients’ OS.

### Neoantigen vaccination elicited stronger neoantigen-specific immune response in RFA+Vac patients

**Figure 3:**
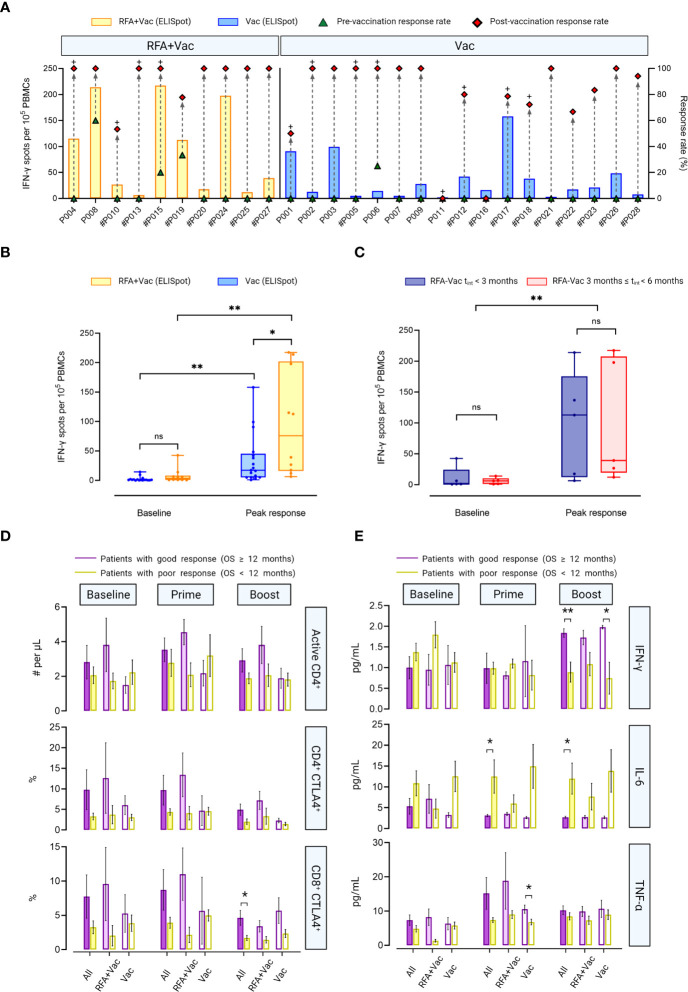
RFA+Vac patients showed better immune responses upon receiving iNeo-Vac-P01. **(A)** *Ex vivo* ELISpot assay results of each patient. Green triangle and red diamond represent the relative response rate, calculated per the ratios of peptides with positive ELISpot reads to all peptides immunized before and after vaccination, respectively. The bar chart shows the spot numbers of the peptide or peptide pool with best reads per 10^5^ PBMCs. # Indicated that peptide pools were used for ELISpot assay for this patient; + indicated that relatively high abundant novel clones of peripheral T cells were detected for this patient after vaccination. **(B**, **C)** Data shown in Box and Whiskers (with minimum to maximum, showing all points and median). **(B)** Baseline and post-vaccination ELISpot spot counts per 10^5^ PBMCs of RFA+Vac (yellow) and Vac (blue) patients. **c**) ELISpot spot counts per 10^5^ PBMCs at baseline and post vaccination of the peak response for RFA+Vac patients with t_int_ < 3 months, or 3 ≤ t_int_ < 6 months. No significant difference was found between the two groups at baseline (blue and yellow); however, the difference was intensified post vaccination, as patients with t_int_ < 3 months showed stronger peak response (red). Baseline response and peak response of all RFA+Vac patients showed statistically significant difference (*p* < 0.0051). **(D)** Flow cytometric analyses of RFA+Vac and Vac patients on T cell subsets. RFA+Vac and Vac patients were further divided into two subgroups: patients with good response (OS ≥ 12 months) and poor response (OS < 12 months); number of active CD4^+^ T cells (# per μL), and proportion of CD4^+^ or CD8^+^ T cells that expressed CTLA4 (%) at baseline, prime phase and boost phase were analyzed, respectively. **(E)** Flow cytometric analyses of RFA+Vac and Vac patients on cytokines including IFN-γ, IL-6 and TNF-α at baseline, prime phase, and boost phase. A t-test analysis was applied to indicate the significance in data difference with a two-tailed *p* value. ns, not significant (p > 0.05); *p ≤ 0.05; **p ≤ 0.01.

Previously, the potentials of iNeo-Vac-P01 vaccination in eliciting T-cell-mediated immune response targeting tumor neoantigens have been demonstrated in patients with advanced solid tumors (10). In this study, T cell activation post neoantigen vaccination was confirmed in 88.89% patients (24/27) by IFN-γ ELISpot assay (**Figure 3A**). All evaluated patients (n=27) showed an increase of spot counts after vaccination (*p* < 0.05), except for patients P006, which might be attributed to the noisy background at baseline. Overall, 77.97% (92/118) individual long peptides and 84.48% (49/58) long peptide pools elicited measurable neoantigen peptide-specific immune response (positive results in ELISpot assay after vaccination) in 21 patients, respectively (**Supplementary Table S6**). TCR sequencing of peripheral T cells at baseline and after vaccination revealed that abundant novel peripheral T cell clones were detected in 60.00% (12/20) patients (**Supplementary Table S7**). These data suggested the effectiveness of iNeo-Vac-P01 in inducing neoantigen-specific T cell activation.

Although RFA+Vac patients showed higher baseline response for IFN-γ ELISpot assay compared to Vac patients, the difference was not significant (*p* = 0.0969). A significant change in spot counts was observed in both RFA+Vac and Vac patients post neoantigen vaccination, indicating the feasibility of personalized neoantigen peptide pools in eliciting robust cellular response. To be noted, RFA+Vac patients exhibited a higher immune response in relative to Vac patients after vaccination with a *p*-value less than 0.05 (**Figure 3B**), showing that RFA prior to vaccination could help enhance antitumor responses drastically. Although not significantly, the time required for RFA+Vac group to witness a peak response was 55.2 days, longer than that of Vac group (51.8 days) (**Supplementary Figure S1**). This phenomenon indicated that the time to obtain an optimal immune response post neoantigen vaccination was probably unrelated to RFA treatment.

To determine the optimal timing of the first vaccination after RFA treatment, RFA+Vac patients were categorized into two subgroups: patients with an RFA-vaccination interval (t_int_) less than 3 months (t_int_ ≤ 3 months) (n=5), and patients with an RFA-vaccination interval between 3 months and 6 months (3 months < t_int_ < 6 months) (n=5). No significant difference in IFN-γ-specific cellular response at baseline was found between these two groups. Differently, patients in RFA-Vac t_int_ ≤ 3 months group showed insignificantly higher peak response upon neoantigen vaccination (**Figure 3C**). A study with larger sample size would be needed to validate whether shorter RFA-vaccination interval [i.e., (t_int_≤ 3 months)] could contribute to a better antitumor immune response.

### Good clinical response was associated with T cell activation and pro-inflammatory cytokine secretion

To further explore the potential reasons for the discrepancy in clinical benefits among patients in this study, patients were further divided into two subgroups according to their clinical response, that are, patients with good response (OS ≥ 12 months) group and patients with poor response (OS < 12 months) group (**Figure 3D** **;** **Supplementary Table S9**). Although not statistically significant, it was observed in RFA+Vac group that good response patients showed more active CD4^+^ T cells, as well as higher proportions of CD4^+^ CTLA4^+^ and CD8^+^ CTLA4^+^ T cells than poor response patients at all time points. Differently in Vac group, poor response patients exhibited insignificantly larger active CD4^+^ T cell numbers than good response patients at both baseline and after prime vaccination It is also noteworthy that in All group (including all patients in both RFA+Vac and Vac groups), good response patients displayed larger number of active CD4^+^ T cells, as well as higher proportion of CD4^+^ CTLA4^+^ or CD8^+^ CTLA4^+^ T cells at each time point. However, only the proportion of CD8^+^ CTLA4^+^ T cells after boost immunization showed significant difference between good and poor response patients (All group) (*p* = 0.0337). These results demonstrated a stronger T cell activation in good response patients than that in poor response patients. In other words, good clinical response was likely to be resulted from efficacious T cell activation by neoantigen vaccination.

Potential biomarkers for clinical response were also investigated in this study, including IFN-γ, TNF-α and IL-6 (**Figure 3E** **;** **Supplementary Table S10**). Similar tendency of the change in IFN-γ secretion post-vaccination was observed compared to our previous study (10). In brief, low IFN-γ secretion was observed at baseline, except for RFA+Vac poor response patients. After boost immunization, IFN-γ secretion in good response patients was upregulated, showing a higher level compared to poor response patients in All group (*p* = 0.0037), Vac group (*p =* 0.0189) and RFA+Vac group (*p =* 0.085), indicating that the efficacy of neoantigen vaccination in upregulating IFN-γ secretion was probably unbiased for Vac and RFA+Vac patients. Overall, the difference in IFN-γ secretion between good response and poor response patients was intensified after vaccination. No disparity in TNF-α expression at baseline was found between good and poor response patients in Vac group, while in RFA+Vac group, good response patients showed a higher bassline TNF-α expression, demonstrating RFA’s efficacy in the activation of immune system. To be noted, the difference in TNF-α expression between good response and poor response patients was first intensified after prime immunization, and then lowered after boost immunization (**Figure 3E**). As a potential tumor growth factor for different cancer types (18), the secretion of IL-6 was also evaluated. Higher level of circulating IL-6 was found in poor response patients compared to good response patients at all time points in all groups, which was in line with previously published studies (10, 19, 20). Also, lower IL-6 expression was found in poor response patients of RFA+Vac group after vaccine administration, indicating that combination therapy may still benefit poor response patients by lowering their circulating IL-6 level.

### Case report of an intrahepatic cholangiocarcinoma patient P015

Good clinical response and immune response were observed in several patients in RFA+Vac group. Here, a case report of a patient who received two batches of customized vaccines, was presented to highlight the importance of applying adjustable and timely personalized treatment for patients to improve their survival. In the case of patient P015, a 56-year-old male diagnosed with adenocarcinoma by right lobe liver lump puncture in August 2017, had multiple liver RFA treatments prior to iNeo-Vac-P01 vaccination. He went through the combinational treatment of Tegafur and Gemcitabine 7 times post-diagnosis, before his first liver RFA treatment in late November 2017. After another liver RFA under the guidance of ultrasound in January 2018, he had Tegafur orally for 2 cycles. Followed by his third liver RFA in late February 2018, he took Apatinib orally for targeted therapy. His most recent RFA prior to neoantigen vaccination was in May 2018, around 2 months before his first dose of iNeo-Vac-P01.

**Figure 4:**
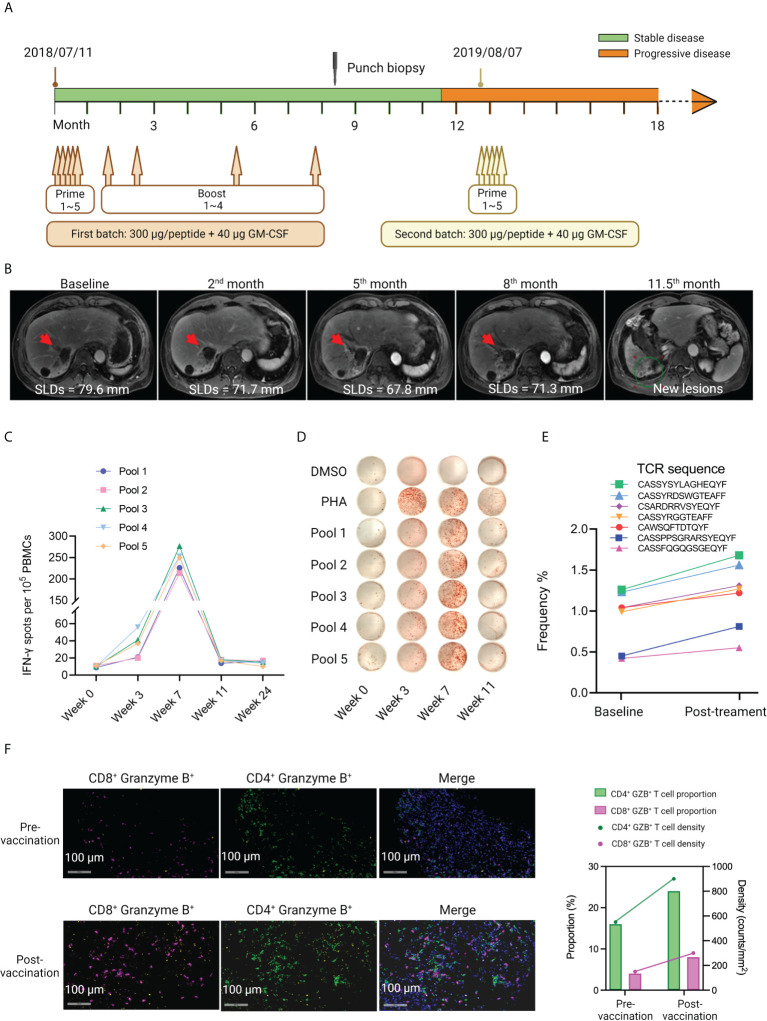
Case report of patient P015, who had multiple RFA treatments prior to vaccination. **(A)** The treatment scheme of patient P015. Two batches of iNeo-Vac-P01 were scheduled for this patient. **(B)** CT scan images of the tumor at different time points during the treatment. The red arrows indicated the corresponding tumor lesion. SLDs: sum of longest diameters. SLDs: sum of the longest diameters. **(C, D)** *Ex vivo* ELISpot assay of IFN-γ^+^ PBMCs during vaccination. **(E)** Increased abundance of peripheral neoantigen-specific T cell clones post vaccination as detected by TCR sequencing. **(F)** Multiplexed IF images of FFPE samples obtained from patient P015 pre- and post-vaccination. Signals of CD8, CD4 and Granzyme B are shown in fuchsias, green and yellow. Double stranded DNA is shown in blue. Shown on the right are the proportion (%) and density (counts per mm^2^) of CD4^+^ Granzyme B^+^ and CD8^+^ Granzyme B^+^ cells.

For patient P015, the first batch of immunization comprising 5 prime doses and 4 boost doses of iNeo-Vac-P01 started from 11^th^ July 2018 (**Figure 4A**). During the first five months post first vaccination, continuous reduction in size of the liver lump was observed, the sum of longest diameters (SLDs) decreasing from 79.6 mm at baseline, to 71.7 mm at the second month and then 67.8 mm at the fifth month, respectively (**Figure 4B**). However, multiple new lesions were observed 11.5 months after first vaccination. Sequencing of his recent biopsy sample enabled the preparation of a new batch of vaccine. The second batch of iNeo-Vac-P01 was delivered to him since 7^th^ August 2019 (**Figure 4A**). It is noted that each peptide pool managed to elicit robust neoantigen-specific T cell responses, and the peak response for each pool was observed 7 weeks post first immunization **(** **Figures 4C, D** and **Supplementary Table S6**). The gradual decline of T cell responses in PHA group at week 3, 7 and 11 in **Figure 4D** was probably resulted from the differences of cell status at these time points. TCR β chain of peripheral T cells was sequenced and the results revealed that the abundance of seven TCR clones considerably increased after vaccination (**Figure 4E**). These data suggested that multiple subsets of T cells with specificities induced by iNeo-Vac-P01 could be successfully activated to kill tumor cells. Both the density and proportion of CD4^+^ Granzyme B^+^ and CD8^+^ Granzyme B^+^ T cells exhibited an increase post-vaccination, indicating the enhancement of the infiltration of T cells into tumor tissues (**Figure 4F**).

Compared to other patients, patient P015 had a relatively long survival time (**Figure 1A**). This could be correlated with the good antitumor immune response, as evaluated by *ex vivo* ELISpot assay. Multiple RFA treatments prior to neoantigen vaccination might contribute to the release of tumor-specific antigens, thereby leading to the immune priming of several neoantigens. Moreover, the timely preparation and delivery of the second batch of iNeo-Vac-P01 after disease progression was also considered beneficial to prolong his survival.

### Combination treatment of RFA and neoantigen vaccination inhibited tumor growth in mice

**Figure 5:**
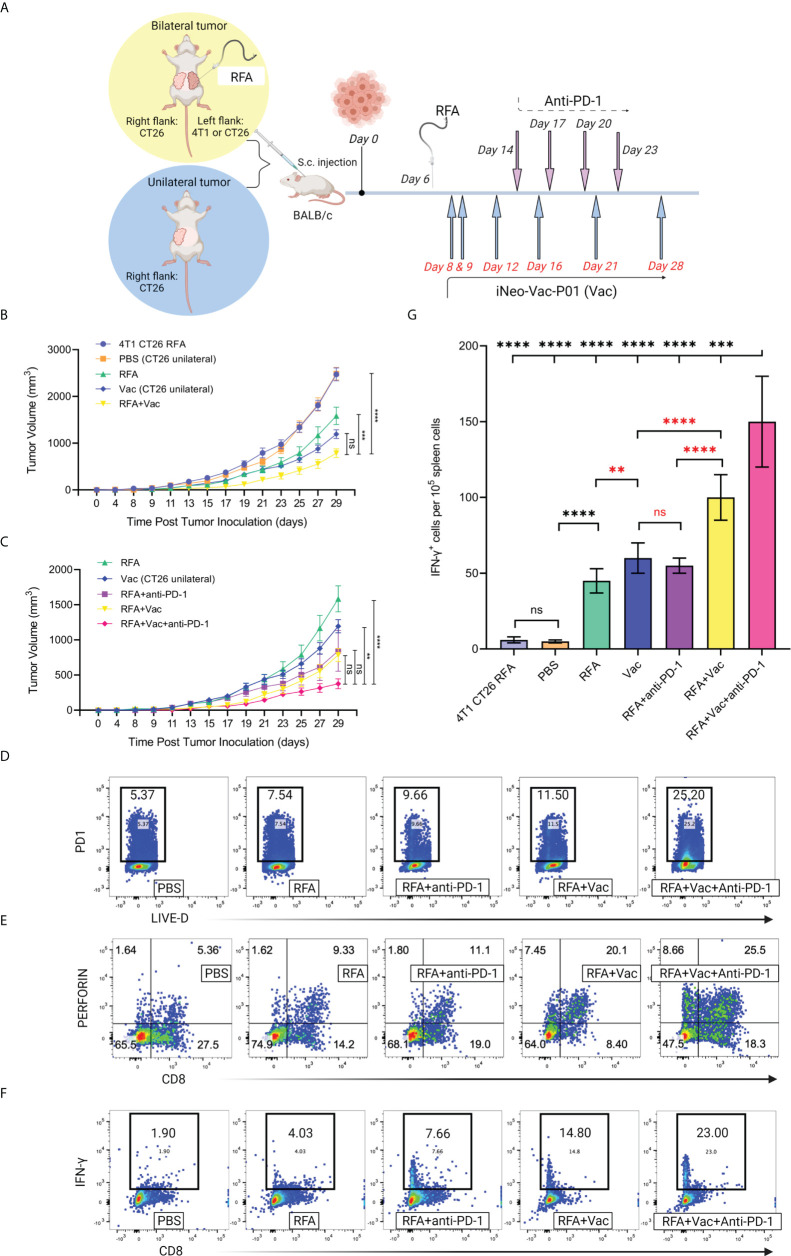
Combination treatment of RFA and neoantigen vaccination inhibited tumor growth and elicited robust local and systemic antitumor immune responses in mice. **(A)** Schematic illustration of the treatment mice received. Mouse models with unilateral or bilateral tumor(s) were established; mice with bilateral tumors received RFA treatment on the left flank tumor. **(B, C)** Tumor growth of mice receiving different treatments. Ablation of an irrelevant tumor (4T1) did not help inhibit the growth of CT26 tumor. Combinational use of RFA and iNeo-Vac-P01 (RFA+Vac) significantly slowed down the growth of CT26 tumor, and the inclusion of anti-PD-1 further enhanced this antitumor effect. **(D)** Flow cytometric analyses of PD-1 expression in tumor tissues (n=3). **(E)** Flow cytometric analyses of multifunctional T cells, gating on CD8^+^ perforin^+^ cells (n=3). **(F)** Flow cytometric analyses of cytotoxic T cells, gating on CD8^+^ IFN-γ^+^ cells (n=3). **(G)** *Ex vivo* ELISpot assay. Spleen cells were collected and re-stimulated by neoantigen peptides. Data shown in Mean ± SD; two-tailed *p* value was obtained through unpaired t test. ns, not significant (p > 0.05); **p ≤ 0.01; ***p ≤ 0.001; ****p ≤ 0.0001.

Inspired by the good clinical and immune response of neoantigen peptide vaccination after RFA treatment amongst patients with different cancer types, mouse models were employed to validate whether a combinational treatment modality incorporating RFA and subsequent individualized neoantigen-based vaccination could generate stronger antitumor effects than either of the single modality alone. As it has been reported that the ablation of a tumor could lead to the shrinkage of another distant tumor (21–23). Thus, to study the synergetic antitumor efficacy of combination treatment and the abscopal effects of RFA, we constructed a murine unilateral CT26 tumor model (unilateral tumor), as well as bilateral tumor models with CT26 or 4T1 tumor implanted on the left flank and CT26 implanted on the right flank (bilateral tumor) (**Figure 5A**).

Compared to the CT26 tumor growth of mice bearing unilateral CT26 tumor that received only PBS (PBS group), mice bearing bilateral heterogeneous tumors (4T1 on the left flank and CT26 on the right flank) showed similar CT26 tumor growth on the right flank after RFA was given on the left flank (**Figure 5B**), indicating no evident antitumor effects. This phenomenon demonstrated that the ablation of an irrelevant tumor 4T1 did not slow down the growth of target tumor CT26. In contrast, slower CT26 tumor growth on the right flank was observed in mice bearing bilateral homogenous CT26 tumors (RFA group) after RFA treatment on the left flank, indicating the abscopal effects induced by RFA were probably resulted from the activated immune cells that could recognize and kill the tumor cells harboring the same antigens as those released from the eliminated tumor cells through ablation. Similarly, when neoantigen vaccines were administered to mice bearing unilateral CT26 tumor (Vac group), evident inhibition of CT26 tumor growth was reported. Compared to mice in both PBS and Vac groups, mice bearing bilateral CT26 tumors receiving combination treatment of RFA and neoantigen vaccination (RFA+Vac group) displayed further inhibition of CT26 tumor growth on the right flank, proving the stronger antitumor efficacy of RFA+Vac combination treatment (**Figure 5B**).

ICIs, especially anti-PD-1 antibodies, have previously shown synergistic effects with RFA or neoantigen vaccination in pre-clinical and clinical studies. Also, CT26 colon cancer cell line has been reported to be sensitive to checkpoint blockade (5, 24). Taken these together, it was hypothesized that in the above mice models, the introduction of PD-1 blockades to RFA+Vac combination treatment (RFA+Vac+anti-PD-1 group) could further inhibit the growth of CT26 tumor on the right flank. As expected, a more significant reduction of CT26 tumor growth on the right flank was observed in the mice of RFA+Vac+anti-PD-1 group, compared with that of mice in RFA, Vac, RFA+anti-PD-1, and RFA+Vac groups (**Figure 5C**). It was most likely that RFA+Vac combination treatment might have upregulated the expression of PD-1 in tumor tissues, turning the “cold” tumor into “hot” tumor, thereby making ICI more efficacious. This hypothesis was validated by flow cytometry to some extent, as higher PD-1 expression in tumor tissues was found in RFA+Vac group (11.50%), compared to RFA+anti-PD-1 group (9.66%), RFA group (7.54%) and PBS group (5.37%). To be noted, the highest PD-1 expression was observed in RFA+Vac+anti-PD-1 group (25.20%) (**Figure 5D**). This finding was also in line with several published studies, where subsets of PD-1^+^ T cells elicited robust neoantigen-specific responses in melanoma (25, 26), suggesting that a stronger TCR signaling was correlated with the addition of anti-PD-1 antibody in the combinational modality. To conclude, the favorable antitumor outcome of RFA plus neoantigen vaccination was probably associated with the enhanced infiltration of activated PD-1^+^ T cells, which could be reinvigorated upon PD-1 blockade, as reported in a previously published study (27).

### Combinational modality elicited stronger local and systemic immune responses in mice

The antitumor functions of cytotoxic CD8^+^ T cells include releasing granzymes to induce the apoptosis of target cells, and/or perforin to generate large transmembrane pores on the membrane of target cells for the diffusion of granzymes (28). Here, the antitumor ability of CD8^+^ T cells was evaluated by flow cytometry. Mice in RFA+Vac+anti-PD-1 group showed the highest proportions of CD8^+^ perforin^+^ T cells (25.50%) and CD8^+^ Granzyme B^+^ T cells (3.36%), followed by RFA+Vac (20.10% and 2.89%), RFA+anti-PD-1 (11.10% and 1.55%), RFA alone (9.33% and 1.11%) and PBS (5.36% and 0.36%) (**Figure 5E** and **Supplementary Figure S2**).

To further explore whether the combination treatment of RFA, iNeo-Vac-P01 and/or anti-PD-1 could mount systemic antitumor responses, mice spleen cells were collected for IFN-γ secretion analyses through flow cytometry. Not surprisingly, combination treatment consisting of all three modalities showed the highest CD8^+^ IFN-γ^+^ T cell proportion (23.00%), followed by RFA+Vac (14.80%), RFA+anti-PD-1 (7.66%), RFA (4.03%) and PBS (1.90%) (**Figure 5F**). Consistent with the flow cytometry results, ELISpot assays for IFN-γ secretion upon *ex vivo* re-stimulation of T cells with selected neoantigen peptides also demonstrated that the combined three modalities had most spot counts amongst all groups, with RFA+Vac taking the second place (**Figure 5G**). Altogether, RFA in combination with iNeo-Vac-P01 could enhance both local and systemic antitumor immune responses, with anti-PD-1 further promoted the antitumor effects.

## Discussion

Considered as a standard local treatment, RFA is widely used for different cancer types. However, RFA alone cannot prevent cancer progression sufficiently, as new remote lesions might appear (29). To date, different modalities including ICIs, TLR9 agonists and c-MET inhibitors displayed their synergies with RFA (5, 30, 31), while no study had been reported to validate the synergies between neoantigen vaccination and RFA. During the past five years, an increasing number of researchers have demonstrated that neoantigen is crucial to the success of cancer immunotherapy; more than 130 neoantigen-targeting clinical trials are currently active and many of these neoantigen-based therapies are being studied in combination with checkpoint modulator. It is likely that personalized neoantigen vaccines could become the turning point in improving the survival of cancer patients in the near future. Although vaccination of neoantigens has been proven to augment local radiotherapy’s antitumor activity in a few studies (32); to our knowledge, our study was the first to investigate the synergy between RFA and neoantigen vaccination. As reported previously, some patients who received RFA before neoantigen vaccination seemed to have relatively long OS, implying that RFA could induce tumor necrosis and thereby releasing tumor neoantigens that acted as pro-inflammatory signals (10). Herein, we sought to explore the rationale of combining peptide neoantigen vaccination with RFA to improve their antitumor effects.

All patients enrolled in our study were late-staged patients with relatively bad prognosis. We selected PFS and OS as the markers to indicate the efficacy of neoantigen vaccine, considering that the overall response rate (ORR) and duration of response (DoR) might not be good indicators for the efficacy of the treatment for late-staged patients. Besides, both PFS and OS have been reported to be good therapeutic efficacy markers for late-stage patients who received immunotherapy or chemotherapy (33–39). Patients in our study displayed comparable or longer mPFS and mOS in relative to patients with the same cancer types in these abovementioned studies. Herein, our patients were retrospectively divided into two groups: one received RFA treatment within 6 months before neoantigen vaccination, the other did not. As expected, patients who received pre-vaccination RFA treatment showed longer mPFS and mOS, which correlated with their stronger immune response at baseline and post vaccination. Based on the clinical response, patients were further divided into two subgroups: patients with good clinical response (OS ≥ 12 months) and patients with poor clinical response (OS < 12 months) to better understand the discrepancy in their clinical response in terms of the differences in T cell subsets as well as cytokine profiles. Similar tendency in IFN-γ, TNF-α and IL-6 expression (pg/mL) was observed, compared to that of our previous published data (16). IFN-γ plays an important role in modulating immune response against cancer. No significant difference in IFN-γ expression was found between RFA+Vac and Vac patients after vaccination, suggesting that the vaccination of personalized neoantigen peptides contributed to most of IFN-γ expression (**Figure 3C**). On the contrary, a larger discrepancy in TNF-α expression at baseline between patients with good response and patients with poor response in RFA+Vac group was observed, in comparison to that of Vac group, suggesting that RFA might have contributed to higher TNF-α expression at baseline. Moreover, after vaccination, IL-6 expression was lower in patients with good response compared to patients with poor response, which was in line with a published study concluding that the elevation of IL-6 correlated with poor survival in pancreatic cancer patients (40). Our result here demonstrated that neoantigen vaccination could downregulate the expression of tumor-promoting cytokines such as IL-6 to help achieve better clinical response. In addition, a larger proportion of activated T cells in patients with good response was found compared to that of patients with poor response upon vaccination (**Figure 3D**). As expected, the differences in the proportions of activated CD4^+^, CD4^+^ CTLA4^+^ and CD8^+^ CTLA4^+^ T cells between patients with good response and patients with poor response in RFA+Vac group were larger than that in Vac group, although not significantly. This not only explained that patients with good response might have undergone strong T cell activation upon vaccination, thereby upregulating CTLA4, the negative regulator of T cell activation, as a halting mechanism; but also suggested that the inclusion of anti-CLTA-4 immunotherapy might further benefit these patients’ clinical response (41). Moreover, patient P015 who received multiple RFA treatments, and two batches of vaccines showed more granzyme B in post-vaccination tumor tissue (**Figure 4F**), indicating the better infiltration activity of T cells upon vaccination. In addition, CD4^+^ T cells amongst all enrolled patients exhibited a significant increase in PD-1 expression after vaccination (**Supplementary Figure S3** and **Supplementary Table S8**), suggesting that the addition of anti-PD-1 may further benefit these patients.

Combination treatment of RFA and neoantigen vaccination was performed in mice to further validate their synergies (**Figure 5**). Mice receiving RFA and neoantigen vaccination showed slower tumor growth than mice receiving single modality alone (**Figure 5B**). Triple therapy consisting of RFA, Vac and anti-PD-1 further inhibited tumor growth and showed stronger immune responses compared to double therapy (**Figures 5C–G**). Highest IFN-γ and perforin expression was found in CD8^+^ cells of the mice receiving triple therapy, respectively (**Figures 5E, F**), suggesting the effective activation of multifunctional T cells by triple therapy. In line with our clinical data (**Supplementary Figures S3**; **Supplementary Figures S4** **;** **Supplementary Table S8**), PD-1 expression in mice receiving RFA+Vac treatment was also found higher compared to mice receiving single modality. To be noted, with the inclusion of anti-PD-1, the expression of PD-1 in triple therapy was the highest. This could be explained by the reinvigoration of a T cell subset with high tumor infiltration ability upon the blockade of PD-1, as discussed earlier in the Results Section. Taken these together, our results indicated that cold tumors could potentially turn into hot tumors when neoantigen vaccination is coupled with RFA. More importantly, the antitumor effects of RFA together with neoantigen vaccination could be further promoted with the blockage of PD-1. Further studies should be conducted to investigate the efficacy of triple therapy combining RFA, neoantigen vaccination and anti-PD-1 in patients with different cancer types.

## Conclusions

It was demonstrated in this study that RFA and peptide neoantigen vaccination could have synergies in combating existing tumors. Patients who received pre-vaccination RFA treatment displayed better clinical response upon vaccination of personalized cancer vaccine. Also, the timing for neoantigen vaccination could be of vital importance to control tumor progression, as patients showing better antitumor responses in this study turned out to be those who received more timely vaccination. With the addition of checkpoint inhibition, the synergies of RFA and neoantigen vaccination were further improved in mice model. Taken these together, we conclude that the combinational use of neoantigen vaccination and RFA may bring about longer OS for patients with a variety of cancer types, with immune checkpoint blockade further enhancing the clinical response.

## Data availability statement

The original contributions presented in the study are included in the article/**Supplementary Material**. Further inquiries can be directed to the corresponding authors.

## Ethics statement

The studies involving human participants were reviewed and approved by Institutional Review Board and Independent Ethics Committee of Sir Run Run Shaw Hospital affiliated with Zhejiang University School of Medicine. The patients/participants provided their written informed consent to participate in this study. The animal study was reviewed and approved by Laboratory Animal Management and Ethics Committee of Zhejiang Chinese Medical University. Written informed consent was obtained from the individual(s) for the publication of any potentially identifiable images or data included in this article.

## Author contributions

Conception and design: YF, FM, SZ, JS, HP, and SC. Collection and assembly of data: SZ, LLiu, DM, XG, QH, QG, HL, and WH. Data analysis and interpretation: FM, YF, SZ, MQ, LLu, LLiu, SY, and NH. Manuscript writing: LLu, NH, and FM. Data presentation and graphics: LLu, MQ, LLiu, SZ, and XZ. Final approval of manuscript: all authors. Accountable for all aspects of the work: all authors.

## Funding

This work was supported by the National Natural Science Foundation of China [grant numbers 81702809 and 81872238], the Medical Science and Technology Project of Zhejiang Province [grant numbers 2016ZDB007 and 2017ZD021] and Joint Funds of the National Natural Science Foundation of China [grant number U20A20409], and partly supported by Natural Science Foundation of Zhejiang Province [grant numbers LY13H160013 and LQ16H160003], Health Commission of Zhejiang Province [grant number 2016KYA115], Medical Science and Technology Project of Zhejiang Province [grant number 2017197380], CSCO Health Project [grant numbers Y-QL2019-0316, Y-MSD2020-0314 and XS022] and Zhejiang Medical Innovative Discipline Construction Project-2016.

## Acknowledgments

We appreciate all authors for contributing to this paper.

## Conflict of interest

FM, SZ, LLu, NH, LLiu, MQ, DM, XG, QH, XZ, and SC are employed by Hangzhou Neoantigen Therapeutics Co., Ltd. FM is employed by company Hangzhou AI-Force Therapeutics Co., Ltd. NH is employed by company Hangzhou AI-Nano Therapeutics Co., Ltd.

The remaining authors declare that the research was conducted in the absence of any commercial or financial relationships that could be construed as a potential conflict of interest.

## Publisher’s note

All claims expressed in this article are solely those of the authors and do not necessarily represent those of their affiliated organizations, or those of the publisher, the editors and the reviewers. Any product that may be evaluated in this article, or claim that may be made by its manufacturer, is not guaranteed or endorsed by the publisher.

## Abbreviations

AE, Adverse effect; DMSO, dimethyl sulfoxide; ICI, Immune checkpoint inhibition; OS, overall survival; PBMCs, Peripheral blood mononuclear cells; PFS, progression-free survival; RFA, Radiofrequency ablation; TCR, T cell receptor; TLR, toll-like receptor.
