# Supplementary material for: Combination treatment of radiofrequency ablation and peptide neoantigen vaccination: Promising modality for future cancer immunotherapy
Source: Front Immunol. 2022 Sep 29;13:1000681. doi: 10.3389/fimmu.2022.1000681 (PMC9559398; doi:10.3389/fimmu.2022.1000681)
Supplement: Supplementary file 1 [file DataSheet_1.docx]

**Supplementary Materials**

**Supplementary Methods**

**Generation of patients-specific neoantigen peptides**

Tumor neoantigens were predicted and prioritized by our in-house bioinformatics pipeline iNeo-SUITE as reported previously [1]. In brief, NGS sequencing data of DNA samples was used to call HLA alleles and somatic mutations for each patient, and candidate peptides were inferred from the detected somatic mutations, and further affinity analysis was done using iNeo-Pred and NetMHCIIpan. Gene expression level was measured using RNA sequencing in cases where RNA samples were available, otherwise it was inferred from TCGA samples of the same cancer type. iNeo-SUITE consists of multiple modules including sequencing reading filtering, genome alignment, mutation calling, HLA typing, MHC-binding affinity prediction, gene expression profiling, vaccine peptide sequence design, as well as peptide sequence prioritization based on its therapeutic potency. FastQC (v0.11.4) was employed for the quality control of sequencing data. Reads with more than 4 N bases or a quality score below than 15 were discarded [2]. Qualified reads were then mapped to the human reference HG38 (Human Genome version 38) by using Burrows-Wheeler Aligner software (BWA, v0.7.12). Subsequently, by comparing to the normal sample, tumor somatic mutations were identified by integrating the mutation calling results from Mutect (v2.0), Varscan 2 (v.3.5.19) [3-6], Strelka (1.0.11) and somatic-sniper (v1.0.5.0). Then, somatic mutation candidates were ranked based on their reliability and manually inspected by Integrative Genomics Viewer (IGV v.1.0.6) according to their alignment profile. Germline mutations in both normal and tumor samples were identified by GATK HaplotypeCaller. The database of Single Nucleotide Polymorphism (dbSNP) (<http://www.ncbi.nlm.nih.gov/snp/>) and 1000 Genome datasets were used to filter out high population frequency (PF) mutations (PF > 1%) from somatic mutation candidates. Next, the mutations were further annotated by Variation Effect Predictor (VEP, ensemble v89) [6-8]. HLA typing and quantification were done by OptiType (v1.3.1), Polysolver (V4), PHLAT (release 1.1) and our in-house software iNeo-HLA using reference sequences from IMGT datasets [7].

Subsequently, the flanking sequence of peptide or the upstream sequence of peptide were extracted from human protein database for single nucleotide mutations or frameshift and stop-loss mutations. To predict the neo-epitopes within those peptides, all possible segments containing mutation-induced amino acids were further extracted with the length ranging from 8 to 16 amino acids (8-11 mer for HLA class I and 12-16 mer for HLA class II). HLA class I neo-epitopes were predicted by in-hosue software iNeo-Pred, a deep-learning and machine-learning integrated predictor trained on datasets from IEDB and mass spectrometry (MS) profiling of HLA ligands. Epitopes with HLA binding affinity were predicted using iNeo-Pred HLA class II neo-epitopes were predicted by NetMHCII-pan (v4.0) according to the manual [9]. After the vaccines were designed based on these candidate peptides using iNeo-Design, the information of mutation, HLA allele, expression and affinity was integrated in iNeo-PRIOR to get an iNeo-Score for the prioritization of the vaccines.

After the identification of all neo-epitope candidates, our in-house software iNeo-PRIOR was used to rank the mutations based on their therapeutic potency, whereas the mutation prevalence, gene expression, affinity change, epitope number, and heterologous level of mutant peptides were taken into consideration. A mathematical formula was designed and applied to integrate all factors to calculate the score for prioritization:

*iNeo_Score =* f_1_(Ag) × f_2_(E) × f_3_ (M_i_) × f_4_ (H) + f_5_ (M_ii_)

*iNeo_Score*, Ag, E and H refer to the score for prioritization, mutation prevalence, average gene expression obtained from TCGA database, and heterologous level of mutant peptide, respectively. M_i_ and M_ii_ stand for the quality index which takes affinity change and epitope number into account for epitopes presented by MHC I or MHC II molecules, respectively. Mutations with top ranking scores were chosen, while other factors such as the reliability of the mutation, as assessed by manual examination and Sanger sequencing, as well as function (whether the mutation was in an oncogenic or cancer-driver gene) were also considered.

Mouse CT26-specific neoantigen identification was performed following the procedures described above, with modifications made to meet specific requirements. For example, MM10 mouse reference genome was used for this purpose when mapping sequencing reads. Secondly, NetMHCpan-4.0 instead of iNeo-Pred was used in predicting peptide affinity. Lastly, MHC allele identification was not included in this process as compared to human data, since it was not necessary.

**T cell receptor (TCR) sequencing**

T cell receptor (TCR) β chain was sequenced for each patient before and after vaccination following steps described previously [1]. RNA extraction of PBMCs was performed using RNeasy Plus Mini Kit (Qiagen). One common forward primer adaptor and one reverse primer corresponding to the constant (C) regions of each TCRα and β were designed to facilitate PCR amplification of cDNA sequences in a less biased manner. Samples were analyzed by high-throughput sequencing of TCR using ImmuHub TCR profiling system at a deep level (ImmuQuad Biotech). Briefly, a 5’ RACE unbiased amplification protocol was used. This protocol uses unique molecular identifiers (UMIs) introduced in the course of cDNA synthesis to control bottlenecks and eliminate the errors of PCR and sequencing. Sequencing was performed on an Illumina HiSeq system with PE150 mode (Illumina). One common adaptor with UMI was added on the 5’ of cDNA during the first-strand cDNA synthesis. One reverse primer corresponding to the constant (C) regions of each of the TCRα and β was designed to facilitate PCR amplification of cDNA sequences in a less biased manner. The UMIs attached to each raw sequence reads were applied for PCR, resulting in sequencing error correction and PCR duplication removing. V, D, J and C segments were mapped with IMGT. CDR3 regions were extracted, and clonotype assembled for all clones. The special nucleotide/amino acid sequences of CDR3 region of TCR β subunit were determined. Those with out-of-frame or stop codon sequences were removed from the identified TCRβ repertoire. We further defined the amounts of each TCRβ clonotype by adding TCRβ clones sharing the same nucleotide sequence of CDR3 region. “New high abundant clones” was defined as the clones that were absent or with a frequency of less than 0.001% of all clones sequenced in the blood sample before vaccination but were with a frequency of no less than 0.5% of all clones sequenced in the blood samples after vaccination. “Clones with considerably increased abundance” was defined as the clones that were with a frequency of less than 0.5% of all clones sequenced in the blood sample before vaccination and increased the frequency by no less than 2% after vaccination. Either of these two circumstances is considered as “relatively high abundant novel clones of peripheral T cells detected after vaccination”.

**Culturing of tumor cells**

CT26 (BALB/c mouse undifferentiated colon carcinoma) and 4T1(BALB/c mouse breast cancer) cell lines purchased from American Type Culture Collection (Manassas, United States) were cultured in complete RPMI-1640 medium (Thermo Fisher, Waltham, United States), supplemented with 10% fetal bovine serum, 2 mM L-glutamine and 1% (v/v) penicillin/streptomycin, and maintained at 37 °C in 5% CO_2_. Cells were not further authenticated but cultured for a limited number of passages. PCR was performed to test whether mycoplasma contamination presented.

**IFN-γ enzyme-linked immunospots (ELISpot) assay**

Peripheral blood (10-30 mL) was collected from each patient at different time points, followed by the isolation of peripheral blood mononuclear cells (PBMCs) by Ficoll/Hypaque density-gradient centrifugation (GE Healthcare). IFN-γ ELISpot assay was performed with human IFN-γ precoated ELISpot kit (DAKEWEI). In brief, 200 µl serum-free medium was added into each well, and the plate was incubated at room temperature for 5-10 minutes before discarding the medium.100 µL cell suspension was added to each well at a density of 2×10^5^ cells per well, followed by adding peptide pools containing 5-10 µg/mL neoantigen peptides, or 2 µg/mL CEF peptide as positive control. The plates were then incubated at 37°C for 16-24 hours, followed by adding 200 µL pre-cooled deionized water into each well to lyse at 4°C for 10 minutes. The plates were washed 6 times before the addition of 100 µL biotin-labeled antibody and then incubated at 37°C for 1 hour. After washing the plates, 100 µL enzyme-labeled avidin working solution was added into each well and incubated at 37°C for 1 hour. AEC solution mix was then added into each well after washing the plates, and the plates were kept in the dark for 25 minutes at room temperature before adding deionized water to stop the reaction. An automatic plate reader with appropriate parameters set beforehand was used to read these plates. Samples with more than 100 spots after noise subtraction (based on negative control group) were considered to give strong positive results, while samples with only 8 to 20 spots were considered to give weak positive results.

**Cytometric analysis of T-lymphocyte activity**

Antibodies were purchased from Biolegend, as shown in Table S0. PBMCs were isolated, and T cells were labeled following the instruction of manufacturer. In brief, the corresponding antibodies were added into an empty flow tube and mixed with 100 µL T cell suspension thoroughly, followed by incubation in the dark for 15 minutes. 2 mL of erythrocyte lysate (Zhejiang Bozhen Biotechnology Co., Ltd.) was then added, and the solution was then mixed and incubated in the dark for 10 minutes. The sample was centrifuged at 500×g for 5 minutes, followed by the removal of 1620 µL of supernatant (440 µL remained). Subsequently, 10 µL of absolute count microspheres was added into the tube and mixed. Cytometric analysis was performed after sample preparation.

**Table S0.** Antibodies for flow cytometry

| Fluorescence | Antibody | Clone |
| --- | --- | --- |
| FITC | CD279(PD-1) | EH12.2H7 |
| PerCP/Cy5.5 | CD4 | OKT4 |
| A700 | CD8 | SK1 |
| BV605 | CD152(CTLA-4) | BN3 |

**Cytometric bead array (CBA) analysis of cytokines**

The concentrations of serum cytokines were measured by CBA following manufacturer’s protocol (Hangzhou Saiji Biotechnology Co., Ltd). Th1/Th2 cytokine kit was applied. In brief, 25 µL of captured microspheres solution was added into a blank flow tube, followed by adding 25 µL microsphere buffer solution. The mixture was incubated in the dark for 30 minutes. Subsequently, 25 µL fluorescence detection reagent and 25 µL serum were added in order. The mixture was vortexed and then incubated in the dark for another 2.5 hours. After adding 1 mL PBS solution, the sample was centrifuged at 200×g for 5 minutes. The supernatant was removed and then 100 µL PBS solution was added to resuspend the sample. Samples were analyzed by a flow cytometer and the data were analyzed using FlowJo V10 software.

**References**

1. Fang, Y., et al., *A Pan-cancer Clinical Study of Personalized Neoantigen Vaccine Monotherapy in Treating Patients with Various Types of Advanced Solid Tumors.* Clinical Cancer Research, 2020. **26**(17): p. 4511-4520.

2. Hundal, J., et al., *Cancer Immunogenomics: Computational Neoantigen Identification and Vaccine Design.* Cold Spring Harb Symp Quant Biol, 2016. **81**: p. 105-111.

3. Chen, F., et al., *Neoantigen identification strategies enable personalized immunotherapy in refractory solid tumors.* J Clin Invest, 2019. **129**(5): p. 2056-2070.

4. Hundal, J., et al., *pVAC-Seq: A genome-guided in silico approach to identifying tumor neoantigens.* Genome Med, 2016. **8**(1): p. 11.

5. Ott, P.A., et al., *An immunogenic personal neoantigen vaccine for patients with melanoma.* Nature, 2017. **547**(7662): p. 217-221.

6. Ng, A.W.R., et al., *In silico-guided sequence modifications of K-ras epitopes improve immunological outcome against G12V and G13D mutant KRAS antigens.* PeerJ, 2018. **6**: p. e5056.

7. Hilf, N., et al., *Actively personalized vaccination trial for newly diagnosed glioblastoma.* Nature, 2019. **565**(7738): p. 240-245.

8. Keskin, D.B., et al., *Neoantigen vaccine generates intratumoral T cell responses in phase Ib glioblastoma trial.* Nature, 2019. **565**(7738): p. 234-239.

9. Andreatta, M., et al., *Accurate pan-specific prediction of peptide-MHC class II binding affinity with improved binding core identification.* Immunogenetics, 2015. **67**(11-12): p. 641-50.

**Supplementary Figures**

**
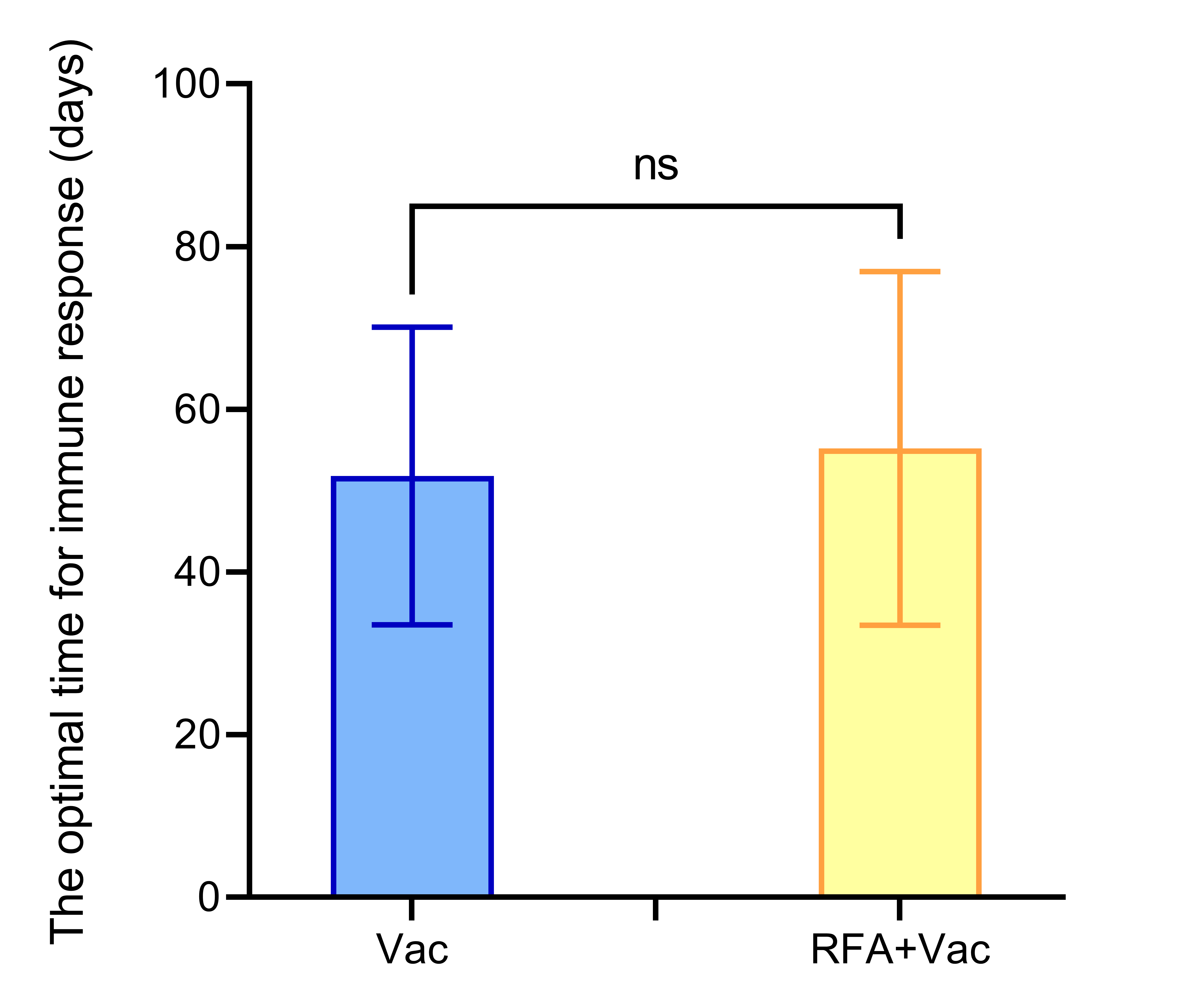
**

**Fig S1.** Optimal time for immune response in Vac and RFA+Vac groups

**
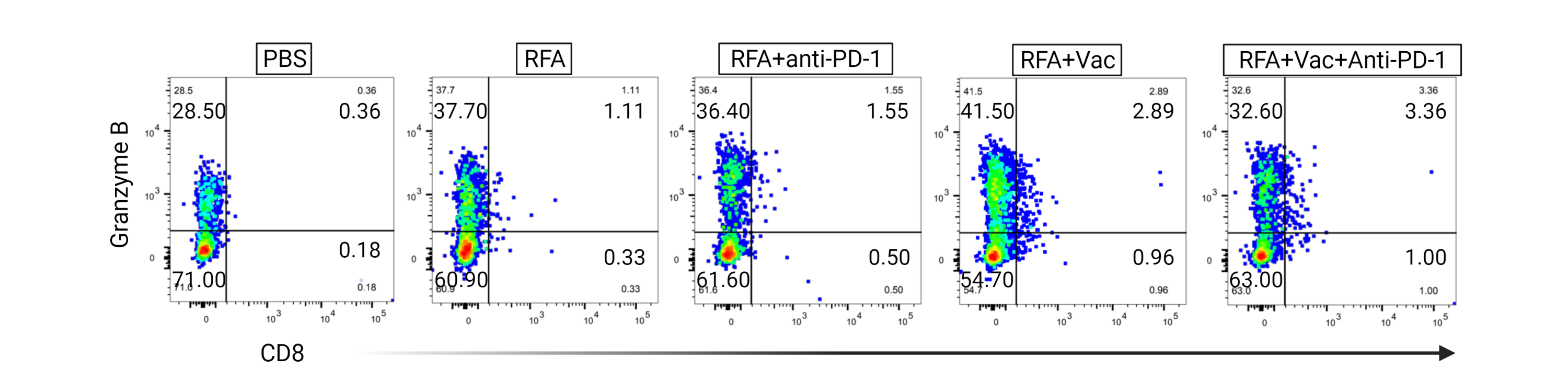
**

**Fig S2.** Flow cytometric analyses of mice multifunctional T cells, gating on CD8^+^ Granzyme B^+^ cells

**
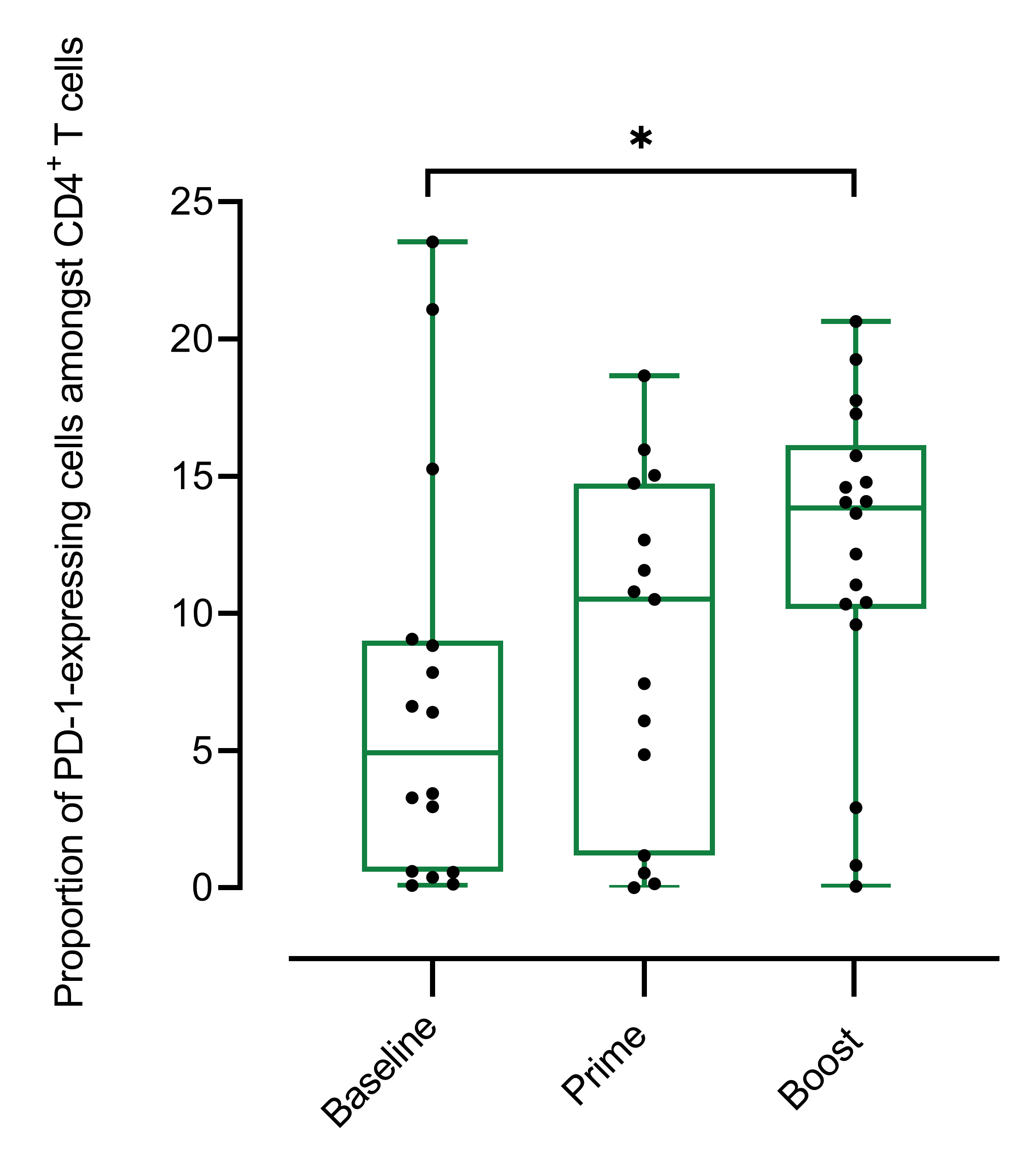
**

**Fig S3.** PD-1 expression in CD4^+^ T cells of all evaluable patients upon vaccination

**
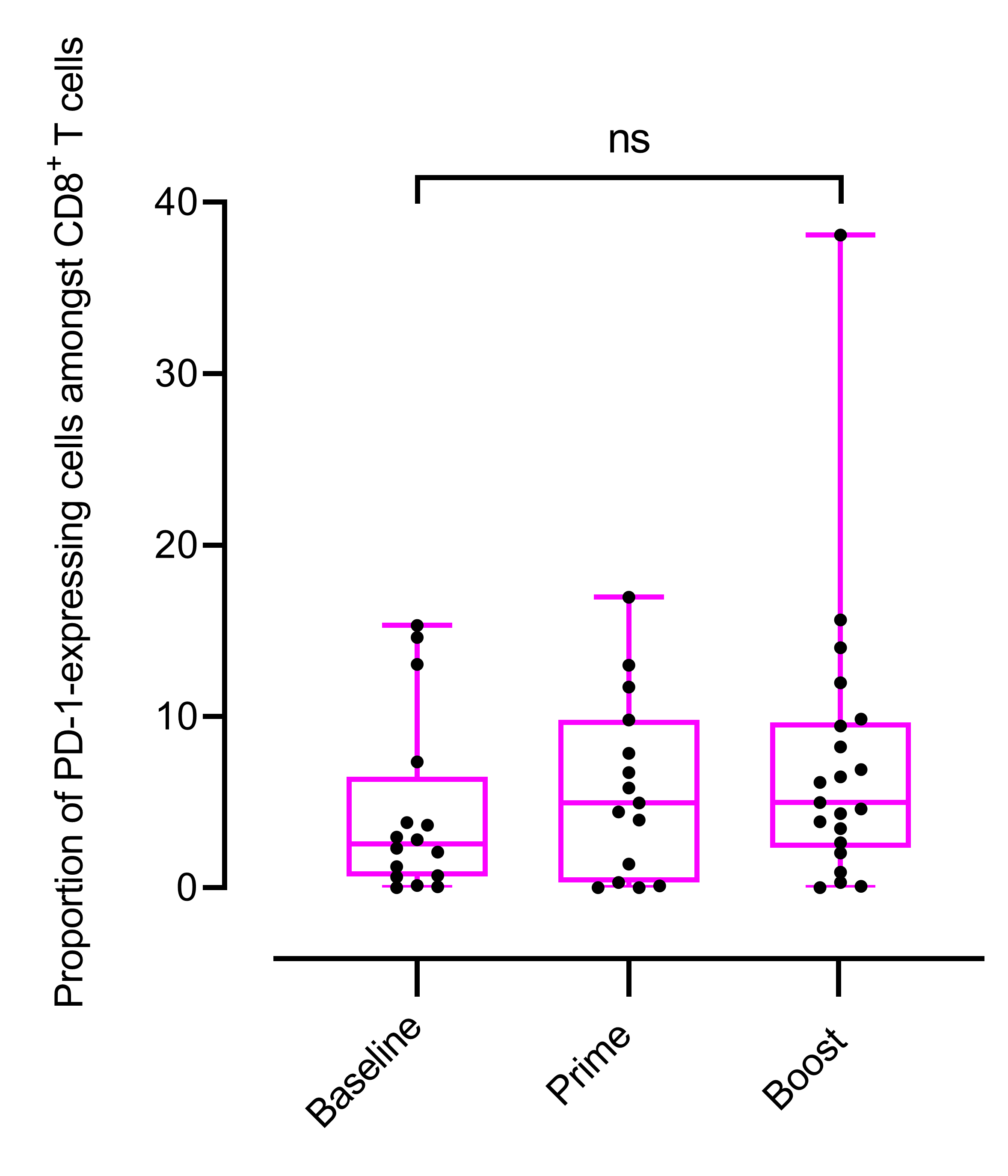
**

**Fig S4.** PD-1 expression in CD8+ T cells of all evaluable patients upon vaccination
